# Danshen decoction in the treatment of heart failure: A systematic review and meta-analysis protocol of randomized controlled trials

**DOI:** 10.1097/MD.0000000000030698

**Published:** 2022-09-16

**Authors:** Mengnan Liu, Raoqiong Wang, Ziyi Li, Maryam Mazhar, Gang Luo, Sijin Yang

**Affiliations:** a Faculty of Chinese Medicine and State Key Laboratory of Quality Research in Chinese Medicine, Macau University of Science and Technology, Macau, Taipa, China; b National TCM Clinical Research Base and Department of Cardiovascular Medicine, the Affiliated Traditional Chinese Medicine Hospital of Southwest Medical University, Luzhou, Sichuan, China; c Institute of Integrated Chinese and Western Medicine, Southwest Medical University, Luzhou, Sichuan, China; d Department of Scientific Research and Department of Neurology, the Affiliated Traditional Chinese Medicine Hospital of Southwest Medical University, Luzhou, Sichuan, China; e School of Clinical Medicine, Southwest Medical University, Luzhou, Sichuan, China.

**Keywords:** clinical research, Danshen decoction, heart failure, meta-analysis, traditional Chinese medicine

## Abstract

Heart failure (HF) is considered the clinical endpoint of all cardiovascular diseases (CVDs). Although a large number of HF drugs and therapies have been developed, it is still need to find therapies with better clinical efficacy and fewer side effects. The purpose of this systematic evaluation is to assess the efficacy and safety of Danshen decoction on HF and the improvement of cardiac function (CF).

**Methods:**

Four databases will be searched to identify any eligible studies, and this protocol does not require ethics committee review as the research is based on published articles. There are no restrictions set for the language, publication date, or status of the study. The clinical effective rate (CER) of HF treatment is considered to be the main result. CF, various serum inflammatory factors, and adverse events were defined as secondary outcomes. When more than 1 article is used to study the changes and results of the same index, we will conduct a meta-analysis. If the heterogeneity is not statistically significant (*P* > .10 or *I*^2^ < 50%), a fixed-effect model will be established to estimate the overall intervention effect. Otherwise, random effect models will be used to provide more conservative results.

**Results:**

The results of this meta-analysis will be submitted to a peer-reviewed journal for publication.

**Conclusion:**

This study will provide reliable evidence-based basis for the clinical application of Danshen decoction in the treatment of HF.

## 1. Introduction

Heart failure (HF) is considered to be the clinical endpoint and the leading cause of death of cardiovascular diseases (CVDs).^[1]^ The World Heart Federation reported that HF causes >10 million deaths every year, that may exceed 20 million people by the year 2030.^[2]^ As a chronic disease, HF is the focus of the Second World Health Revolution. Despite years of research and development, diuretics, cardiotonics, and vasodilators remain the mainstream treatment options for the HF. However, there are certain limitations associated with these therapies. First, these drugs interfere and disturb the homeostatic state of the body. Second, various drug metabolites undergo slow metabolism and excretion leading to their accumulation in the body and ultimate toxicity. Moreover, these drugs are unable to completely cure the disease. With people’s growing desire for a healthy and better life, traditional Chinese medicine (TCM) as an alternative medicine in the prevention and treatment of HF is becoming more popular. The basic and clinical research related to TCM has also been widely concerned by the cardiovascular community of scientists and clinicians.^[3]^

TCM has been inherited and applied for >2000 years and its clinical efficacy has been widely recognized. In all the patients who have been treated for HF, >71.2% patients preferred integrative medicine (IM), 18.74% patients preferred TCM and 10.04% chose western medicine (WM) as a preferred therapeutic method.^[4]^ TCM has a long history of treating HF. It has unique advantages in protecting cardiomyocytes, dredging the cardiovascular system, and inhibiting the inflammatory response. Danshen decoction is composed of *Salvia miltiorrhiza Bge.*, *Amomum villosum Lour*, and *Santalum album L*., and is widely used in the treatment of CVDs and digestive system diseases.^[5]^ In recent years, a large number of preclinical (in vivo/in vitro) experiments and clinical observation studies have proved the therapeutic efficacy of Danshen decoction in the treatment of HF. The pharmacological mechanisms of Danshen decoction include enhancing myocardial cell function, lowering blood pressure, lowering blood lipids, and reducing myocarditis reaction, etc.^[6]^ However, systematic evaluation and review of the clinical treatment of Danshen decoction is insufficient, leaving objective and quantitative evaluation indicators of Danshen decoction to be inadequate. Therefore, evidence-based studies are urgently needed to demonstrate its efficacy and safety.^[7]^

This meta-analysis will search all the studies related to Danshen decoction, formulate inclusion-exclusion criteria, and screen out all the eligible clinical studies.^[8]^ Afterwards, the Revman V.5.4 software will be used for systematic reviews, summaries and meta-analysis of clinical studies. The ability to improve CF and the effect of reducing serum diagnostic indicators such as brain natriuretic peptide (BNP), N-terminal pro-B type natriuretic peptide (NT-proBNP), hypersensitive C-reactive protein (hs-CRP), etc, were used as indicators to evaluate the therapeutic effect of Danshen decoction. Finally, the credibility of the article will be improved by reasonable statistics and correct meta-analysis results, using the chi-square test. The results of this study will provide more updated and comprehensive evidence for clinical decision-making, providing a reference for the follow-up study of Danshen decoction in the treatment of HF.

## 2. Methods

This study is registered on the International prospective register of systematic reviews (PROSPERO), and the ID of the registered study is CRD42022351918. This protocol adheres to the Preferred Reporting Items for Systematic Reviews and Meta-Analyses Protocols (PRISMA),^[9]^ and the specific content of this protocol is shown in the PRISMA-P checklist.

### 2.1. Study selection

#### 2.1.1. Inclusion and exclusion criteria for clinical RCTs.

Relevant RCTs will be selected according to the following inclusion and exclusion criteria. There are no restrictions on the language, publication date, or status of the study.

#### 2.1.2. Inclusion criteria.

Clinical studies related to Danshen decoction. It must be related to various CVDs. The patients either have first diagnosis of HF or the clinical manifestations of HF. It must include but not be limited to CER (clinical effective rate) and any of the following indicators: cardiac color Doppler ultrasound, BNP, etc. HF classification or quantitative indicators of HF must be included.

#### 2.1.3. Exclusion criteria.

The first diagnosis has nothing to do with CVDs. It is not a clinical control experiment or the control group of the experimental group is not clear. The experimental data is not clearly stated or the results are wrong.

#### 2.1.4. Inclusion criteria for patients.

Patients with the first diagnosis of HF, and CVD patients whose CF indicators meet the diagnostic criteria of HF will be included, regardless of age, race, nationality, and basic disease history.^[10]^ According to the clinical guidelines and literature references, the following conditions can be diagnosed as HF: HF has typical symptoms and signs: such as exertional, or nocturnal paroxysmal dyspnea and other different manifestations; patients are often accompanied by lower extremity edema, fatigue and other symptoms, signs include pulmonary rales, lower extremity edema, jugular vein filling, positive hepatic jugular reflux sign, etc. Abnormal cardiac color Doppler: mainly ejection fraction < 50%. The serum markers of HF increased: BNP ≥ 35 pg/mL, NT-proBNP ≥ 125 pg/mL.^[11]^

#### 2.1.5. Inclusion criteria for experimental interventions.

Experimental interventions include control group treatment and experimental group treatment. The control group is treated with conventional HF treatment, and the experimental group is treated with Danshen decoction combined with CT (conventional treatment). For Danshen decoction treatment, the daily Danshen decoction dose is 100 to 400 mL, which is determined by the physician according to the patient’s condition. While using Danshen decoction for treatment, it does not rule out the use of any other nondrug TCM therapies, including *TCM syndrome differentiation*, *QiGong*, *Tai Chi*, *Acupuncture*, *Cupping*, *Moxibustion*, and *Massage*, because Danshen decoction is often prescribed in TCM hospitals, and TCM adjuvant therapy is usually the basic treatment of HF.^[12]^

#### 2.1.6. Inclusion criteria for comparator interventions.

Control interventions included no treatment, placebo, or routine treatment. The drugs, dosage, frequency, and duration of routine treatment will not be limited. If available, the following comparisons will be considered: Comparison of Danshen decoction alone with no treatment. Use Danshen decoction and placebo alone. Comparison between Danshen decoction alone and routine treatment. Comparison between Danshen decoction combined with CT and CT. Comparison between Danshen decoction combined with CT and placebo plus CT.

#### 2.1.7. Inclusion criteria for outcomes.

##### 2.1.7.1. Primary outcomes.

CER is defined as the main result of this meta-analysis. The objective evaluation of the effectiveness of a treatment for the disease after treatment includes 5 grades: clinical cure, significant improvement, mild/moderate improvement, ineffectiveness, and deterioration. It can be evaluated by different types of clinical symptom scales. The nimodipine method is usually used to calculate CER. CER = (basic cure + significant improvement + improvement) number of cases/ total number of cases × 100%.^[13]^ CER is the simplest, most intuitive, and most comprehensive evaluation index to measure a treatment method. We will judge whether Danshen decoction has a curative effect according to the actual situation of CER in the control group and the experimental group.^[14]^

##### 2.1.7.2. Secondary outcomes.

CF, BNP, etc, were identified as secondary outcomes. CF includes but is not limited to LVEF, LVEDD, LVESD, etc. The improvement of CF is a significant sign that all CVDs are controlled and the treatment have good curative effect, and it is also an important turning point for the recovery of HF. Other inflammatory indicators that have a therapeutic effect on HF, such as BNP, NT-proBNP, hs-CRP, etc, will be a better choice if they can be included in the secondary indicators.

### 2.2. Data sources and search strategy

#### 2.2.1. Electronic sources and search strategy.

**Figure 1.**
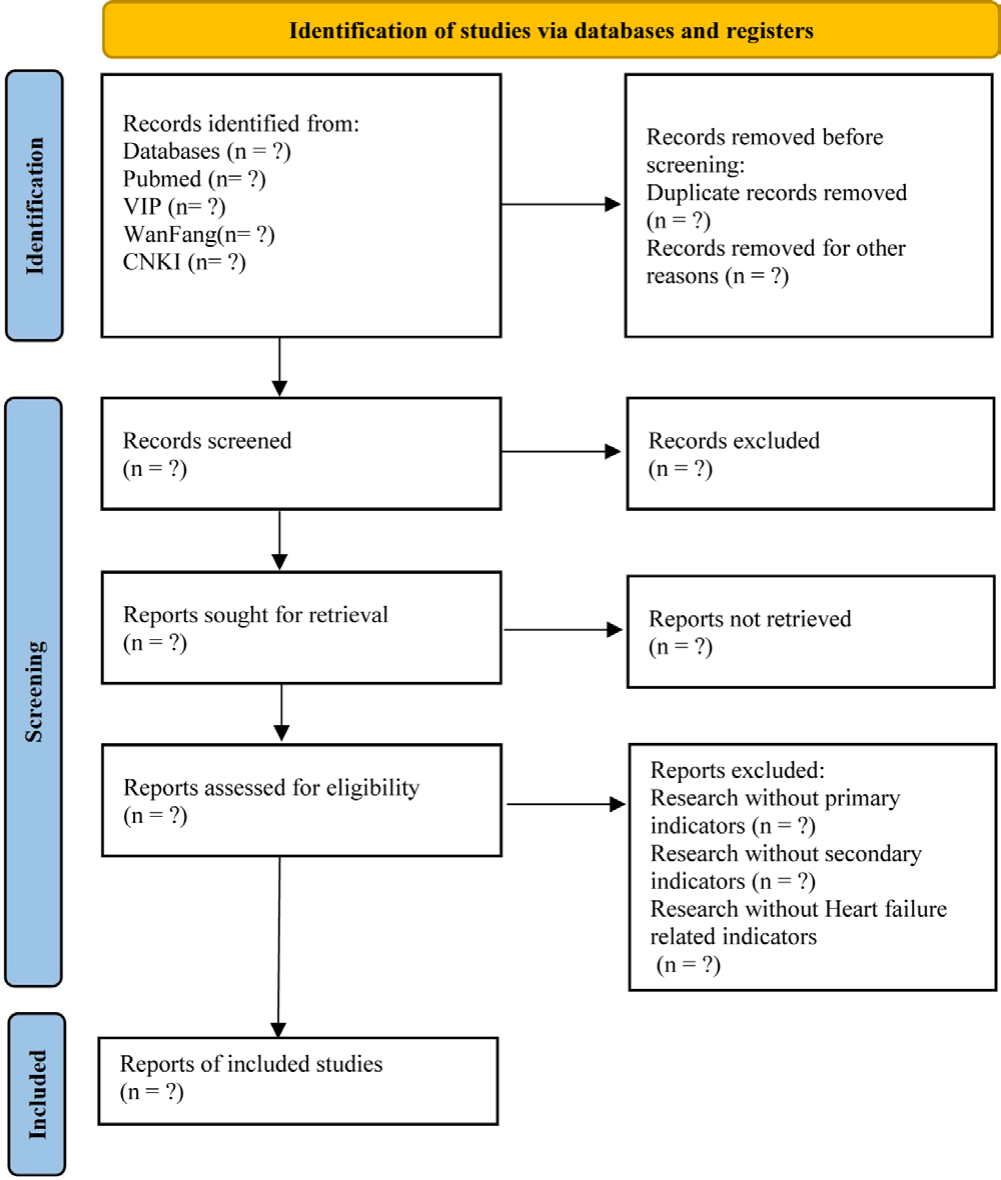
Identification of studies via databases and registers.

The clinical RCTs and meta-analyses for treating HF will be searched in the relevant database from their inception to August 10, 2022, including PubMed, EMBASE, Cochrane Library, Chinese BioMedical Literature Database, China National Knowledge Infrastructure, and Wan Fang Database, and Chinese Scientific Journal Database. There will be no restriction on language, publication date or status. Taking PubMed as an example, the detailed search strategy is shown in Table 1. The literature screening based on PRISM is shown in Figure 1.

**Table 1:** Search strategy in PubMed.

	Search strategy in PubMed	
Search	Query	Items found
#1	Heart Failure[MeSH Terms]	
#2	Heart failure[Title/Abstract]	
#3	Cardiac failure[Title/Abstract]	
#4	Heart decompensation[Title/Abstract]	
#5	Heart dysfunction[Title/Abstract]	
#6	Cardiac dysfunction[Title/Abstract]	
#7	Ventricular dysfunction[Title/Abstract]	
#8	Heart dificiency[Title/Abstract]	
#9	Cardiac dificiency[Title/Abstract]	
#10	Heart insufficiency[Title/Abstract]	
#11	Cardiac insufficiency[Title/Abstract]	
#12	#1 OR #2 OR #3 OR #4 OR #5 OR #6 OR #7 OR #8 OR #9 OR #10 OR #11	
#13	Danshen Decoction[MeSH]	
#14	Danshen Decoction[Title/Abstract]	
#15	Danshen Yin[Title/Abstract]	
#16	#13 OR #14 OR #15	
#17	Randomized Controlled Trials as Topic[Mesh]	
#18	Randomized Controlled Trial[Publication Type]	
#19	Controlled Clinical Trial[Publication Type]	
#20	Equivalence Trial[Publication Type]	
#21	Randomized controlled trial[Title/Abstract]	
#22	Random Allocation[Mesh]	
#23	Double-Blind Method[Mesh]	
#24	Single-Blind Method[Mesh]	
#25	Clinical Trial[Publication Type]	
#26	Research Design[Mesh]	
#27	Placebos[Mesh]	
#28	Placebo[Title/Abstract]	
#29	Random[Title/Abstract]	
#30	Trial[Title]	
#31	#17 OR #18 OR #19 OR #20 OR #21 OR #22 OR #23 OR#24 OR #25 OR #26 OR #27 OR #28 OR #29 OR #30	
#32	Systemic Review[Publication Type]	
#33	Systemic review[Title/Abstract]	
#34	Systemic literature review[Title/Abstract]	
#35	Meta Analysis[Publication Type]	
#36	Meta analysis[Title/Abstract]	
#37	Meta-analysis[Publication Type]	
#38	Meta-analysis[Title/Abstract]	
#39	Pooled analysis[Title/Abstract]	
#40	Consensus Development Conference as Topic[Mesh]	
#41	Consensus Development Conference[Publication Type]	
#42	Consensus development conference[Title/Abstract]	
#43	Expert consensus[Title/Abstract]	
#44	Practice Guideline as Topic[Mesh]	
#45	Practice Guideline[Publication Type]	
#46	Practice guideline[Title/Abstract]	
#47	Cochrane database systemic review[Title/Abstract]	
#48	Evidence-based Medicine[Mesh]	
#49	Evidence-based medicine[Title/Abstract]	
#50	Best practice[Title/Abstract]	
#51	Evidence synthesis[Title/Abstract]	
#52	Synthesis analysis[Title/Abstract]	
#53	#32 OR #33 OR #34 OR #35 OR #36 OR #37 OR #38 OR #39 OR #40 OR #41 OR #42 OR #43 OR #44 OR #45 OR #46 OR #47 OR #48 OR #49 OR #50 OR #51 OR #52	
#54	#12 AND #16 AND #31 AND #53	

#### 2.2.2. Other resources and search strategy.

We will manually search the list of references for major studies and related reviews to identify other references. We will also visit “ClinicalTrials.gov,” the WHO international clinical trial registration platform, and the website of the China clinical trial registry to identify more ongoing or unpublished studies.^[15]^

#### 2.2.3. Data extraction and management.

Two investigators will independently extract the following information: General information (title, first author, year of publication). Experimental design characteristics (experimental design scheme, experimental grouping, randomization principle, blind method, sample size). Patient characteristics (age, nationality, first diagnosis, basic diseases, complications). Clinical observation characteristics (control group intervention, experimental group intervention, drug name, drug dose, frequency of use, treatment course). Outcomes (primary and secondary outcome indicators, outcome indicator units, outcome evaluation methods, blind outcome evaluation methods, adverse reactions). If necessary, we will contact the authors of the included studies to provide further details or clarifications.

#### 2.2.4. Assessment of the risk of bias.

The Jadad scale tool will be used to check the methodological quality of each reference included in the trial by 2 investigators.^[16]^ Four aspects were included: random sequence generation, randomized hiding, blind method, and withdrawal. Three levels will be used to evaluate the quality of the method: “high compliance” (2 points), general compliance (1 point), and “noncompliance” (0 points). If necessary, differences will be discussed with other researchers to reach consistent conclusions. For each included study, the risk of bias in each field will be classified as low, high, or unclear. If a study describes it as an RCT but does not report the randomization method, we will try to contact the author to provide further details or clarification. If the information about the sequence generation process is insufficient to judge “low risk” or “high risk,” the risk of bias will be rated as “unclear.” The overall risk of bias in the study is estimated to be low only when all 4 aspects are rated as low risk of bias. When there are differences in the risk of bias between studies, we will try to analyze the influencing factors of the risk of bias.^[17–20]^ The deviation risk map and deviation risk summary will be generated by Revman V.5.4. Any differences will be resolved through discussion and analysis with the third investigator.

#### 2.2.5. Management of missing data.

If possible, we will contact the original author to request to check the data. Only reasonable and correct data can be used in the primary analysis.

#### 2.2.6. Assessment of heterogeneity.

Statistical heterogeneity across the studies will be tested using Chi-square test. When the *P*-value of chi-square is <.05, the heterogeneity will be considered statistically significant. When *I*^2^ > 50%, exploratory sensitivity or subgroup analysis will be carried out to determine the possible cause.

#### 2.2.7. Subgroup analysis.

If possible, subgroup analysis will be conducted for the study if it shows significant heterogeneity and subgroup analysis will be performed based on the following variables: The first diagnosis of the patient. Nonacute and acute diseases of patients. A Chi-square test will be carried out for the difference in intervention effect in each subgroup.

#### 2.2.8. Data synthesis.

When an indicator has more than 1 clinical study and has comparable primary and secondary indicators, we will conduct a meta-analysis of this indicator. If there is no heterogeneity, a fixed-effect model will be established to estimate the overall intervention effect. If there is heterogeneity in various clinical studies in the indicators, the random effect model will be used to analyze the results. When multiple intervention groups are used in the study, we will group them according to the characteristics of each intervention group and conduct a subgroup analysis. All statistical analyses will be performed by Revman V.5.4 software. Statistical significance was defined as *I*^2^* *< 50%. If the meta-analysis is not feasible, we will provide a narrative description of the results.

#### 2.2.9. Sensitivity analysis.

When the data are correct, in the case of extreme worst and best cases, sensitivity analysis will be used to evaluate the impact of abnormal data on the final results.

#### 2.2.10. Reporting bias.

Inverted funnel diagrams will be used to determine the publication bias with regards to primary outcome and secondary outcomes, and related adverse events. If there are >10 trials, a formal statistical test will be conducted for the detection of possible publication bias by investigating funnel plot asymmetry.

## 3. Discussion

This meta-analysis will comprehensively review the efficacy and safety of Danshen decoction on HF. Meanwhile, the shortcomings and prospects of this study will also be explained in detail. Evidence from this meta-analysis may benefit the clinical treatment of HF, and it will also contribute to the promotion of TCM and the formulation of clinical guidelines. However, the various biases and high heterogeneity of outcome indicators encountered in the study may pose potential challenges to the quality of this study.

## Amendments

If the protocol is modified, the change, the rationale, and the date of any amendment will be described in the final report.

## Acknowledgments

We would like to thank Maryam Mazhar for the language revision.

## Author contributions

Conceptualization & Data curation: M.L., Z.L., and R.W.

Formal analysis & Investigation: M.M.

Visualization: Z.L.

Writing—original draft: M.L. and Z.L.

Writing—review & editing: G.L. and S.Y.
